# Current Proceedings in the Molecular Dissection of Hepatocellular Adenomas: Review and Hands-on Guide for Diagnosis

**DOI:** 10.3390/ijms160920994

**Published:** 2015-09-02

**Authors:** Diane Goltz, Hans-Peter Fischer

**Affiliations:** Institute of Pathology, University Hospital of Bonn, 53127 Bonn, Germany; E-Mail: hans-peter.fischer@ukb.uni-bonn.de

**Keywords:** liver tumor, hepatocellular adenoma, molecular pathology, genetics

## Abstract

Molecular dissection of hepatocellular adenomas has brought forward a diversity of well-defined entities. Their distinction is important for routine practice, since prognosis is tightly related to the individual subgroup. Very recent activity has generated new details on the molecular background of hepatocellular adenoma, which this article aims to integrate into the current concepts of taxonomy.

## 1. Clinical Background

**Figure 1:**
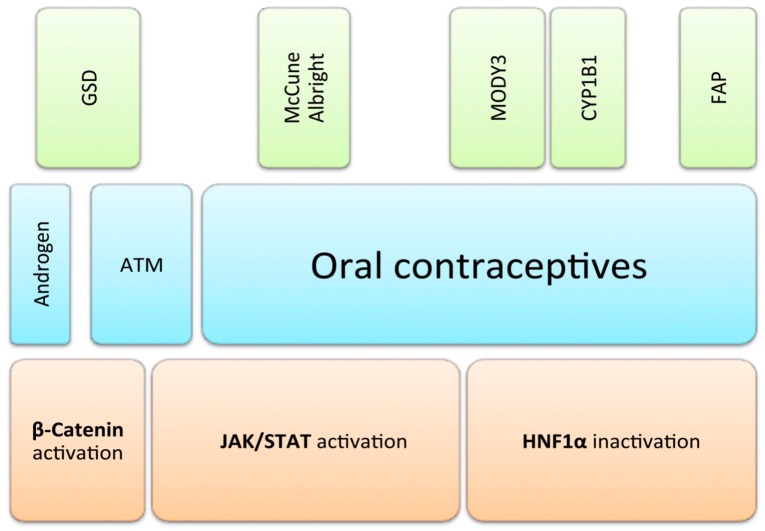
Risk factors of hepatocellular adenomas (HCA). Various genetically-defined predisposing factors are depicted in green. Blue bars represent environmental factors frequently associated with the development of HCA, second to none the use of oral contraceptives in females. Orange boxes in the lowermost line represent three distinguishable molecular pathways, which define the main subgroups of HCA. GSD: glycogenosis; FAP: familiar adenomatous polyposis; ATM: alcohol, tobacco, metabolic syndrome; JAK: Janus kinase; STAT: signal transducer and activator of transcription; HNF1α: hepatocytic nuclear factor 1 α.

Hepatocellular adenomas (HCA) are rare, benign liver tumors in young- and middle-aged people, frequently occurring in women with a long lasting history of oral contraceptives [1]. Obesity, vascular diseases, elevated androgen levels, tobacco, and alcohol abuse, as well as syndromic diseases (McCune-Albright syndrome, glycogen storage disease type 1a, and maturity-onset diabetes of the young (MODY) type 3, familiar adenomatous polyposis (FAP)) add to the known risk factors for HCA [2,3,4] (Figure 1).

Clinically, HCAs may indolently present as a palpable tumor or they may ultimately lead to hemorrhage and shock [5]. Malignant transformation to hepatocellular carcinoma (HCC) is a sporadic event, which is generally recognized to occur in 4.2% to 10.6% [6,7]. Of note, prevalence of malignancy is ten times higher in affected men compared to their female counterpart [7,8].

Therapeutic management of HCA is not yet standardized and stands outside the scope of this article. In a nutshell, however, first-line therapy of small lesions in females comprises withdrawal of medical products containing estrogen. For males, in general, and for females with tumors exceeding a cross-section diameter of 5 cm, surgical resection is recommended [9].

**Figure 2:**
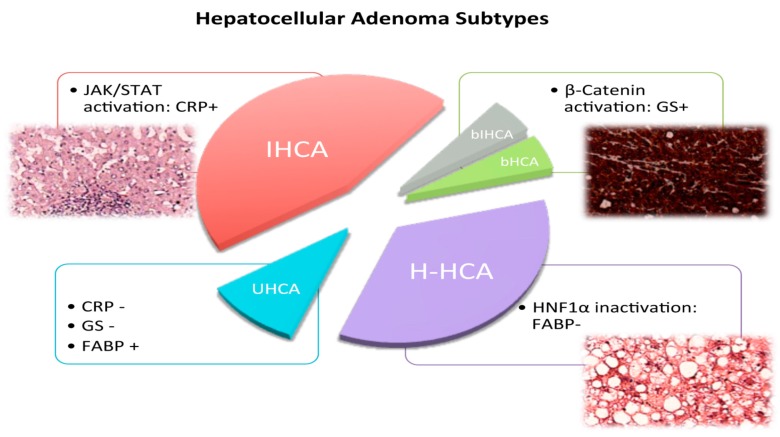
Hepatocellular Adenoma Subtypes. Three main subgroups are currently distinguished among hepatocellular adenomas (HCA). The inflammatory group (IHCA) accounts for 45% of HCA and is characterized by an activation of the JAK/STAT pathway, resulting in an inflammatory phenotype with sinusoidal dilation and inflammatory infiltrates (red). Diagnosis is based on immunohistochemical CRP expression. The subgroup of β-catenin activated HCA is split into two: solely β-catenin activated HCA (bHCA, green) and tumors that in addition to β-catenin activation display inflammatory phenotypes (bIHCA, gray). Together, they account for 10% of HCA. In most cases, diagnosis is rested on diffuse immunohistochemical GS expression. The second most frequent subgroup is characterized by a loss of HNF1α and comprises 35% of HCA (H-HCA, purple). Phenotypically, these tumors display steatosis and are found to display negative FABP immunohistochemical staining. The fourth subtype to be depicted is the yet unclassified group of HCA (UHCA, blue). CRP: C-reactive protein; GS: glutamine synthetase; FABP: fatty acid binding protein 1.

During the past decade, Zucman-Rossi’s group has been achieving tremendous progress in characterizing HCA on a molecular basis. They defined four subcategories of HCA substituting three groups with genetically-defined tumorigenesis. Mutations inactivating hepatocytic nuclear factor 1 α (HNF1α) are responsible for 35% of HCA (H-HCA) [10,11,12], mutations activating β-catenin result in β-catenin activated HCA (bHCA) in 10% of affected patients [9], mutations activating the JAK/STAT3 pathway lead to inflammatory HCA (IHCA), accounting for 45% of HCA [5,13]. The remaining, yet unclassified tumors, lack the latter characteristics [5] (Figure 2). In 2010, the French classification found its way into the WHO Classification of Liver Tumors; since then, pathologists have been applying it on small biopsies and resection specimens.

## 2. Hepatocytic Nuclear Factor 1 α (HNF1α) Inactivated Hepatocellular Adenomas (HCA)

**Figure 3:**
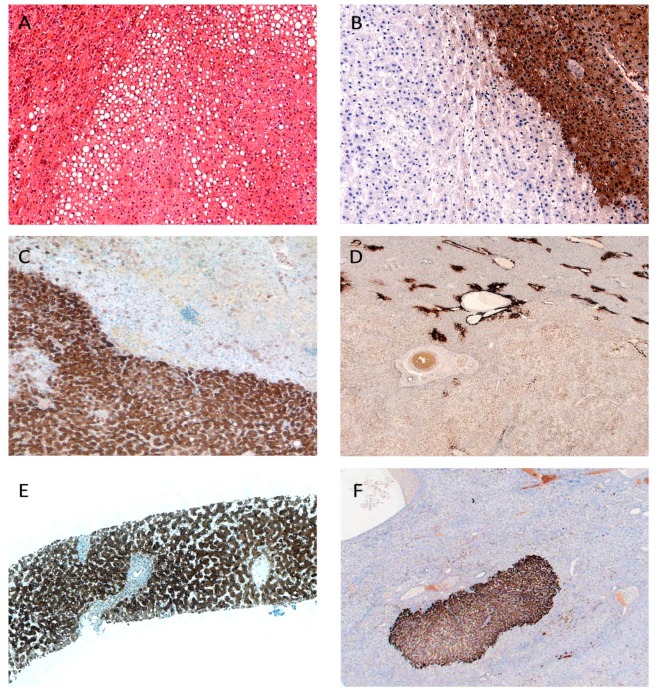
Immunohistochemical features of hepatocellular adenoma subtypes. Typically, HNF1α inactivated HCA (H-HCA) display plurivesicular steatosis as seen on this biopsy (**A**); together with loss of FABP in immunohistochemical stains on the resection specimen of the same tumor (**B**); bHCA feature diffuse and intense GS staining if a *CTNNB1* Exon 3 mutation is present (**C**); staining is only faint in cases with *CTNNB1* Exon 7/8 mutation (**D**); inflammatory HCA are characterized by strong and uniform immunohistochemical CRP staining (**E**) together with sinus dilation and inflammatory infiltrates. Frequently, the vicinity of an inflammatory HCA shows small neoplastic foci of CRP staining consistent with microadenomas (**F**). CRP: C-reactive protein; GS: glutamine synthetase; FABP: fatty acid binding protein 1.

HNF1α inactivated H-HCAs historically were the first subgroup to be defined on a genetic basis and hold bi-allelic inactivating mutations of the *HNF1α* gene [11]. Most alterations to be detected are missense mutations and frame shifts [14]. The breakdown of HNF1α leads to transcriptional down-regulation of downstream fatty acid binding protein 1 (FABP) and UDP-glucuronosyltransferase-2B7 (UGT2B7). Contrasting juxtaposition of downstream protein expression in tumor and normal liver is well established for the routine diagnosis of H-HCA. In particular, low FABP levels in H-HCA tissues are easily assessable by immunohistochemistry. This approach achieves a sensitivity and specificity of 100% for the diagnosis of H-HCA even on small biopsies [9,15]. Typically, resected tumors show irregular outlines and plurivesicular steatosis [5,16], a morphological feature that is due to the paramount importance of HNF1α for the physiological handling of glucose and fatty acid in hepatocytes (Figure 3A,B). Main consequences of its loss include repression of gluconeogenesis, activation of glycolysis, and dysregulation of fatty acid synthesis [17,18,19].

While the vast majority of H-HCA develops spontaneously, susceptibility is fundamentally raised in a group of patients with MODY3 who harbor certain heterozygous germ-line mutations of *HNF1α* leading to a severely impaired function of the encoded protein [12,20]. Individual cases with compound heterozygosity in the *HNF1α* and *histone deacetylase 4* (*HDAC4*) genes have been described in this context [14]. Essentially, all males that present with H-HCAs feature a MODY3 as a predisposing factor [8]. Besides germinal mutations in *HNF1α* itself, H-HCA manifestation is tightly linked to oral contraception in women who display heritable variations in Cytochrome P450 1B1 (CYP1B1), catalyzing drug metabolism of steroids [21]. On the whole, genotoxic activity of estrogen metabolites may play a major role in the development of H-HCA even in females without metabolic disorders [12,22]. Of note, H-HCA do not occur in patients with glycogen storage disease (GSD), neither are they associated with *CTNNB1*, *IL6ST*, *GNAS*, and *STAT3* mutations [9]. In contrast to the other genetically-determined subtypes of HCA, DNA hypermethylation resulting in down-regulation of genes particularly affects protein expression in this entity [14]. It is generally accepted that H-HCAs hold the lowest rate of malignant transformation among all subtypes; therefore clinical surveillance seems warrantable in small tumors [23].

## 3. β-Catenin Activated HCA

β-catenin activated HCA (bHCA) is special in that it more frequently occurs in men than any other subtype of HCA. Traditionally, it is said to be tightly linked to the usage of anabolic-androgenic steroids, but it also strikes patients treated with Danazol for anemia [8,24]. Malignant progression is frequently observed [25], Zucman-Rossi *et al.* observed concomitant HCC in as many as 46% of bHCA [13]. Since malignant foci may have a subtle phenotype, strictly speaking, a benign diagnosis can only be made in conjunction with a resection specimen.

On a molecular basis, bHCAs are characterized by somatic mutations in the *CTNNB1* gene coding for β-catenin, impairing its phosphorylation and subsequent degradation [26]. In consequence, β-catenin overwhelms the cytosol and accumulates within the nucleus, thereby effectuating its power as a cotranscription factor and thus constitutively stimulating the Wnt/β-catenin pathway [27,28]. Thereby, a bunch of target genes is overexpressed, amongst them *GLUL* coding for glutamine synthetase (GS) [29]. Physiologic regulation of the Wnt/β-catenin axis is of paramount importance for the maintenance of cell-cell interactions and zonation of liver tissue [30].

*CTNNB1* exon 3 mutations are the classical hotspots of driving mutations in both HCA and HCC [27]. However, recent data show that substitutions at β-catenin in exon 7 and at codon 335/387 in exon 8 are associated with Wnt/β-catenin activation as well, albeit with a lesser intensity [14]. While the mutagenic role of *CTNNB1* exon 3 mutations is well established, recent research has produced conflicting data concerning *CTNNB1* exon 7/8 mutations. Rarely, HCC are affected by *CTNNB1* exon 7/8 mutations [31], however, functional *in vitro* data suggest that these mutations are of minor importance for the malignant progression of liver tumors [14]. The *CTNNB1* mutational status is reflected in the intensity of GS staining in HCA: *CTNNB1* exon 3 mutations are associated with intense and diffuse GS staining (Figure 3C) while *CTNNB1* exon 7/8 mutations may display only faint and patchy staining (Figure 3D) [14,32]. In consequence, diffuse tumorous GS staining is 100% specific, but achieves a sensitivity of just 75% [33]. Nuclear β-catenin staining is highly specific as well, but may only be present in individual nuclei of bHCA [16], and a negative nuclear staining event in no way rules out a bHCA. Therefore, it is regarded as an unreliable surrogate-staining event for *CTNNB1* mutations of either type in HCA, due to an altogether low sensitivity [16]. Recently, organic anion transporting polypeptide 1B3 (OATP1B3) expression has been associated with an activation of the Wnt/β-catenin pathway in HCC [34,35]; however, its role in HCA has not been elucidated yet.

Half bHCAs combine genetic events activating Wnt/β-catenin signaling plus inflammatory phenotypes and, therefore, are classified dually and shortened bIHCA (see below) [14]. On the contrary, β-catenin activating mutations are absent in H-HCAs [9].

In current practice, intense and diffuse GS staining either in conjunction with nuclear β-catenin retention or not, pinpoints bHCA/bIHCA with high risk of malignant transformation. However, the specificity of GS as a surrogate marker of *CTNNB1* mutations is limited in that its expression is sensitive to pathophysiologic changes in liver perfusion as well as gall metabolism, especially in small biopsies [32]. In doubtful cases, a molecular approach should be chosen to assess *CTNNB1* mutations. Once an activating β-catenin mutation is diagnosed, the patient should proceed to surgical resection of his/her tumor as far as possible. The resection specimen should be rigorously examined for atypia, invasive foci, or other hallmarks of malignant transformation before the diagnosis of bHCA is established. However, there is on-going debate when to classify these tumors as well-differentiated HCC [36]. For β-catenin mutated tumors displaying focal atypia without blunt invasiveness, Bedossa *et al*. proposed the new category “well-differentiated hepatocellular neoplasm of undetermined malignant potential (HUMP)” [37], however, the neologism seems not to be well-accepted yet [38]. In cases of diagnostic difficulty, molecular analysis again might serve as a loophole: Recent data suggest that *CTNNB1*-mutations without any signs of atypia are very early events in the malignant transformation of liver nodules, that need to be accomplished by a second hit, the *telomerase reverse transcriptase* (*TERT*) *promoter* mutation, to carry forward malignancy [38], thereby *TERT promoter* mutation takes on a gatekeeper role in the formation of HCC developed from HCA [39]. HCA with foci of malignancy revealed 44% of concomitant *TERT promoter* mutations, while *TERT promoter* mutations were absent in thoroughly benign HCA [40].

## 4. Inflammatory HCA

Forty-five percent of HCA display an inflammatory phenotype and can be summarized within the heterogeneous subgroup of IHCA. They share the non-communicable risk factors obesity, alcohol intake, and tobacco [5,9], which are also encountered in hepatocellular carcinoma [41]. Sixty percent of IHCAs are characterized by somatic gain of function mutations in the *interleukin-6 signal transducer* (*IL6ST*) gene locus encoding the oncogenic gp130 protein, the co-receptor and signal transducer of the IL-6 receptor. Frequently, mutations encompass a hotspot locus in exon 6, only rarely exon 10 is affected by in-frame deletions [14,42]. Mutations in the tyrosine kinase catalytic domain of *fyn-related kinase* (*FRK*), a member of the Src kinase family, occur in 10% of IHCA and result in phosphorylation of the signal transducer and activator of transcription 3 (STAT3) [14]. Five per cent encompass somatic mutations in *STAT3* itself [43]. Another 5% comprise somatic mutations (most comprising missense mutations) in the *GNAS* complex locus coding for, among other proteins, the G-protein α subunit [3]. At last, Pilati *et al.* encountered individual cases that harbor driving mutations within the pseudo-tyrosine-kinase domain of *Janus kinase 1* (*JAK1*) accompanied by auto-phosphorylation of the mutant protein [14]. Of note, activating *JAK1* mutations have previously been found in approximately 9% HCC, the majority of which occur in the pseudo-tyrosine-kinase domain as well [44].

All genomic alterations have in common that they positively target on the JAK-STAT-pathway eventually leading to the distinctive phenotype of IHCA including inflammatory infiltrates, dysplastic arteries, and sinusoidal dilation [13,45]. As a characteristic feature, IHCA with *GNAS* mutation tend to display enhanced fibrosis [14]. All listed somatic gene mutations are vastly mutually exclusive driving mutations in IHCA [14]. Therefore, bridging the 18% gap of unknown drivers in IHCA will be subject to future research [14].

The main diagnostic characteristic of IHCA, namely activation of the JAK-STAT-pathway, can be immunohistochemically proven by a homogenous, non-mosaic-like expression of C-reactive protein (CRP) and Serum amyloid A (SAA) in all tumor cells (Figure 3E). Thereby, tumorous tissue generally displays a more intense staining than the surrounding liver tissue due to an autonomously-activated JAK-STAT-pathway. According to our own observations, however, the intensity of CRP/SAA staining is not as relevant as the uniform pattern of CRP/SAA labeling among tumor cells.

In IHCA, malignant transformation has been related to the presence of β-catenin activation in bIHCA, therefore, precise data are lacking.

## 5. Unclassified HCA

One tenth of HCA cannot be classified according to the previously-introduced subtypes. By definition, they are neither Wnt/β-catenin nor JAK/STAT-activated, plus they lack HNF1α inactivation. Recent insight has put forward epigenetic alterations to potentially explain the remaining 10% of HCA [14]. HCA may show silencing in *p16^INK4a^* and *p14^ARF^* gene promoters by hypermethylation, however, a relation to genetically-defined subgroups has yet to be established [46].

## 6. Adenomatosis

By definition, adenomatosis is diagnosed when more than 10 individual nodules are encountered in one liver [47]. It is associated with several genetic backgrounds. Patients with MODY3 might be at risk of developing different and multiple subtypes of HCA. As already mentioned, severe impairment of HNF1α predisposes for H-HCA and often leads to multiple nodules of that kind. Of note, H-HCA and IHCA/bIHCA may abreastly exist in one liver [48].

GSD is an autosomal recessive disorder of glucose metabolism in which the formation of HCA is regarded as the major cause of morbidity [49]. Interestingly, patients with GSD never develop H-HCAs, and the spectrum of genetic alterations leading to IHCA, bHCA, and combined bIHCA differs from sporadic forms, leading to an enhanced risk of malignant transformation in GSD associated adenomas [2,50]. Up to 40% of IHCA have not been allocated a mechanism of JAK/STAT3 activation [2]. Moreover, the share in *CTNNB1* exon 7 mutations is fairly elevated in GSD associated HCA [2] and has been associated with HCC [31].

Adenomatosis may sporadically arise in patients with FAP, especially when exposed to environmental cofactors e.g. treatment with anabolic androgens [51]. The *adenomatous polyposis coli* (*APC*) gene serves as a tumor suppressor and its mutation forms the molecular basis of FAP. Germ-line mutations lead to truncated APC proteins [52], and in conjunction with a second hit, FAP-related hepatocellular adenomas have been associated with bi-allelic inactivation of the *APC* gene [53,54]. Although loss of APC protein leads to an up-regulation of β-catenin [55], recent comprehensive analysis of FAP related liver nodules revealed that only 4% of lesions presented immunohistochemical features corresponding to β-catenin activation [54]. Moreover, activating mutations of the *CTNNB1* gene were excluded in the majority of FAP-related neoplasms [55]. Of note, FAP-related adenomas have frequently been shown to harbor an inactivation of the *HNF1α* gene resulting in H-HCA [56,57,58].

## 7. Differential Diagnosis of HCA and Hepatocellular Carcinoma (HCC)

From the diagnostic point of view, the development of HCC from pre-existing HCA is a rare event accounting for at least 4.2% of HCA across all entities [6]. Malignant transformation may arise from well-defined macroscopically-detectable malignant nodules within an HCA or, alternatively, derive from microscopically-small malignant foci [7]. Notwithstanding all recent molecular progress, the diagnosis of hepatocellular malignancy basically relies on morphological criteria. A thorough analysis of histological criteria defining malignancy remains the first step in the diagnosis of a well-differentiated hepatocellular tumor. According to Sempoux *et al*. [25], reticulin staining is highly recommended to identify focal malignant transformation within an HCA. Localized loss of the reticulin network may militate in favor of malignancy, particularly if it is combined with structural or cellular atypia (e.g. pseudoglandular formation, nuclear hyperchromasia) [25]. Immunohistochemical staining of CD34, the onco-fetal proteoglycan Glypican-3 (GPC3) and heat shock protein 70 (HSP70) may add substantial arguments for the presence of malignancy as well. CD34 highlights the capillarized endothelial cells of the tumor’s microvascular bed. A thoroughly-positive staining may be associated with malignancy, but it is also found in HCA [59]. In non-cirrhotic livers, positive GPC3 or positive HSP70 staining is very reliable to identify malignant foci with a specificity of 100% [60], however, they achieve a sensitivity of just 43% and 46%, respectively.

In that context, it seems of relevance that immunohistochemical markers and molecular analyses used to subcategorize HCA may be misleading in the differential diagnosis between a benign and malignant hepatocellular neoplasm in a non-cirrhotic liver. In light of frequent *CTNNB1* mutations in both HCA and HCC, GS achieves a sensitivity of 80% and a specificity of just 50% for the detection of malignancy [60]. CRP expression is frequently found in HCC as well, and it is associated with adverse outcomes [61]. In analogy, 11% of HCC display a significant down-regulation of FABP [62] (Supplemental Figure S1).

## 8. Future Perspectives

Most sporadic cases of HCA do not present within the macroscopic context of adenomatosis, however, clinical series revealed multiple immunohistochemically and molecularly detectable foci of JAK/STAT activation within the proximity of IHCA (Figure 3F) [14,63]. Patients that present with such field effects may be future candidates for pharmacologic intervention. Targeted therapies may go for the deprivation of signaling pathways on the basis of individual genetics of the neoplastic lesion. Src inhibitors, in particular Dasatanib, proven to even out the JAK/STAT activation induced by STAT3 and FRK mutants, might mark the first step towards personalized care in HCA [14,43].

As a matter of importance, the gatekeeper TERT is sought-after as a therapeutic target. However, anti-TERT vaccines failed to induce a T-cell response in patients with advanced HCC [64]. Future studies are owing to elucidate if anti-TERT vaccines might be preventive for progression in patients with inoperable high-risk HCA.

## 9. Conclusions

**Figure 4:**
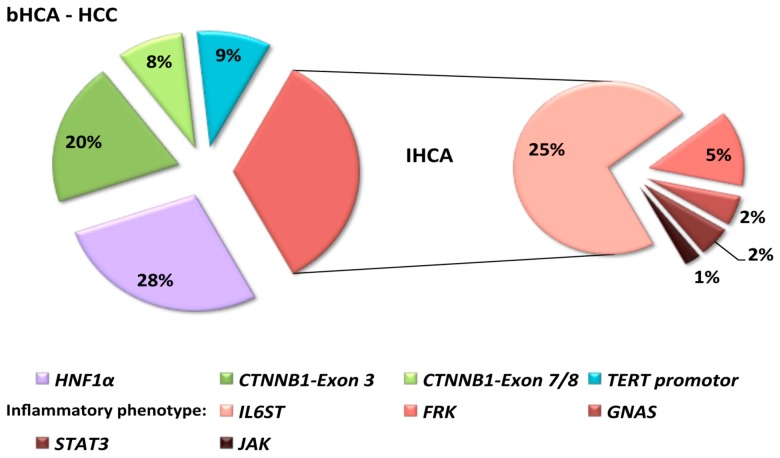
Spectrum of molecular alterations in well-differentiated hepatocellular lesions. The figure is based on 250 hepatocellular tumors investigated by Pilati *et al*. [14]. The spectrum of lesions covers classical hepatocellular adenomas (*n* = 223) and hepatocellular carcinomas (HCC) developing in hepatocellular adenomas (*n* = 27); subtyping referred to 73 HNF1α inactivated tumors (2 with features of malignancy), 76 β-catenin activated tumors (bHCA), thereof 41 with inflammatory features, 77 inflammatory HCA (IHCA), and 23 unclassified HCA (UHCA) [14]. *TERT promotor* mutations were restricted to HCCs. *HNF1α:* *hepatocyte nuclear factor 1α*; *CTNNB1*: *gene coding for β-catenin*; *IL6ST:* *interleukin-6 signal transducer*; *FRK*: *fyn-related kinase*; *STAT3*: *signal transducer and activator of transcription 3*; *JAK: Janus kinase*.

Since the past decade, a great deal of molecular work has been shining a light on benign hepatocellular tumors and has let on about an unexpected genetic diversity. Thereby, a number of genetic events have been described (Figure 4), among which *CTNNB1*-mutations deserve special attention pointing to tumors with high-risk of malignant progression. In this connection, the role of *TERT promoter* mutations triggering malignancy in HCA is unique. Future research will reveal whether modern translational studies will have an impact on clinical care and the evolution of new pharmaceutics.

## Supplementary Materials

Supplementary materials can be found at http://www.mdpi.com/1422-0067/16/09/20994/s1.
