# Supplementary material for: Current Proceedings in the Molecular Dissection of Hepatocellular Adenomas: Review and Hands-on Guide for Diagnosis
Source: Int J Mol Sci. 2015 Sep 2;16(9):20994–1007. doi: 10.3390/ijms160920994 (PMC4613237; doi:10.3390/ijms160920994)
Supplement: Supplementary file 1 [file ijms-16-20994-s001.pdf]

Supplementary Information

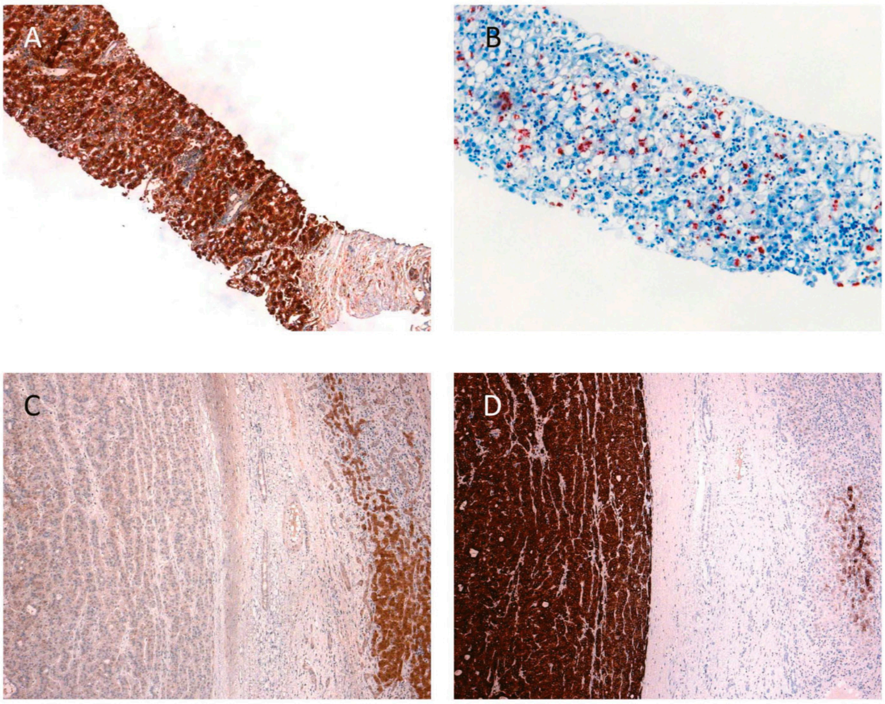

Figure S1. Immunohistochemical staining in hepatocellular carcinoma (HCC). (**A**) Steatohepatitic features in HCC are frequently associated with diffuse and intense CRP expression; (**B**) Ubiquitin staining highlights Mallory Denk bodies underscoring the steatohepatitic nature within the same tumor; (**C**) This HCC displays a loss of FABP (left half) compared to the surrounding liver tissue; and (**D**) the same tumor shows strong GS staining. CRP: C-reactive protein; FABP: fatty acid binding protein; GS: glutamine synthetase.
